# ‘Care literacy’ in super aging Japan

**DOI:** 10.12688/openreseurope.13853.1

**Published:** 2021-07-27

**Authors:** Hiroko Costantini, Misato Nihei, Tomoyuki Ueno

**Affiliations:** 1 Observatoire sociologique du changement (OSC), SciencesPo, Paris, 75337, France; 2 Institute of Population Ageing, University of Oxford, Oxford, OX2 6PR, UK; 3 Department of Human and Engineered Environmental Studies, Graduate School of Frontier Sciences, University of Tokyo, Kashiwa City, Chiba prefecture, 277-8563, Japan; 4 Institute of Gerontology, The University of Tokyo, Tokyo, 113-0033, Japan; 5 Department of Rehabilitation, Koga/Bando Clinical Education and Training Center, Faculty of Medicine, University of Tsukuba, Tsukuba City, 305-8577, Japan

**Keywords:** Care literacy, Community-based integrated care systems, Aging in place, Inclusive society, Community health, Care communications, Japan

## Abstract

Accentuated by the ongoing coronavirus disease (COVID-19) pandemic, the change in Japan to community-based health and care services for older adults indicates an urgent need to enhance and spread citizens’ understanding of care. This is a broader notion of care that incorporates conditions within the community to support the inclusion of older adults, involving not only those older adults receiving care and their direct providers of care, but also others in the community who are involved in the daily lives of these older adults. To underpin such a broader notion of care across citizens, this paper proposes ‘care literacy’ as a novel analytical concept, defined as the knowledge and capabilities that enable people in need of care to live their daily lives in the community and facilitate potential health and care solutions. Reflecting the interconnection of health and care and rooted in the local context, care literacy underpins aging by enabling this involvement of the broader community, and is disseminated through media and grassroot activities.

## Introduction

In the midst of the coronavirus disease (COVID-19) pandemic, the United Nations announced the ‘ Decade of Healthy Ageing’, an action plan calling on member nations to ‘improve the lives of older people, their families, and the communities in which they live’. The pandemic has reaffirmed the vulnerability of older people and demonstrated the urgent need to enhance and spread ‘care literacy’ to ordinary citizens. Such ‘care literacy’ underpins a broader notion of care that incorporates conditions within the community to support the inclusion of older adults, involving not only those older adults receiving care and their direct providers of care but also others in the community who are involved in the daily lives of these older adults. To address the gap in understanding of this broader notion of care, this paper puts forward the understudied concept of ‘care literacy’, defined as *the knowledge and capabilities that enable people in need of care to live their daily lives in the community and facilitate potential health and care solutions*. Beyond the pandemic, the broader framing of including communities in the context of healthy aging raises the importance of community-wide care literacy, which is particularly important for societies undergoing unprecedented population aging. Japan, at the forefront of this shift with the world’s oldest population, is in the process of transforming health and care services for older adults by developing community-based integrated care systems (hereafter CbICS), which provides an appropriate context in which to address this issue.

A baseline understanding of care stems from considering not only older adults and their families, but also what people in their community, for instance, neighbors, shopkeepers, transport operators would need to know to facilitate older people continuing to live in their homes while pursuing healthy aging. An integrated perspective of health care for older adults inextricably raises the issue of place, as for them it is in their lived space that the interweaving of care assistance with daily life and health services occurs ^1– 3^, and it also raises the issue of the involvement of the local community in such systems. Indeed, integrated care systems tend to emphasize empowerment of those who use the system – such as care receivers and their family members – and more generally the involvement of citizens in care ^4^. In Japan, the introduction of the CbICS model began in 2005, with the aim of establishing such systems in each of around 1,700 local areas by 2025, when the post-war baby-boomer generation will reach the age of 75. Due to be fully developed by 2040, when four in ten people will be aged 65 or over, each local municipality designs, governs, and operates the CbICS so as to tailor to community needs given local geographic characteristics. In turn, for the notion of care literacy to take seed with the community and be actualized, care literacy needs to be conceptually rooted in the care practices of the place, encompassing aspects of health, daily living and social interactions.

Furthermore, the adaptation of care in response to COVID-19 points to the holistic perspective necessary to address care systems in order to support older adults in living their daily life and enable healthy living. With an appropriate understanding of care literacy being increasingly relevant to a wider segment of society, the need to reach a mass audience points to a potential complementary role for media and grassroot activities at different geographic scales, so as to address care integration with health issues and location specificities.

Accordingly, the development of the CbICS model offers an opportune lens for considering the issue of care literacy as a potentially significant element in aging in place. While various aspects related to care literacy have been addressed, ‘care literacy’ as a concept has not been developed in spite of its importance. Hence, this paper discusses the value of understanding care literacy, as a concept that is grounded in care practices and rooted in local context, thus underpinning aging in place by enabling involvement of the broader community, and that is disseminated through media and grassroot activities.

## The need for care literacy in community-based integrated care systems

Building an understanding of how CbICS is intended to operate and how it currently functions would assist carers and care receivers in undertaking the proactive role envisioned for them in the context of care. However, there is evidence that significant gaps remain in terms of the public’s awareness and understanding of CbICS. For instance, in Saitama Prefecture, part of Greater Tokyo, a large-scale local government survey revealed that 45% of citizens were unaware of CbICS as of 2018. Part of the complexity in achieving the necessary public awareness stems from the fact that, on the supply-side, CbICS require significant local coordination and collaboration for a variety of services ^5, 6^, with a corresponding need for the education of professional staff ^7^ and novel approaches to inter-service management ^8^. Notably, because health and care services involve elements that have different service catchment areas, such as hospitals, day-care facilities and meal home-delivery services, achieving integration, such as the necessary governance and delivery mechanisms, necessarily implicates different geographic scales ^9^. Further, local differences matter, such as the feasibility of home visits in sparsely populated areas and fostering community engagement in urban areas ^10^. The necessary focus on the complexities of integration may, however, leave a user-centric perspective in the background ^11, 12^.

The CbICS model, nonetheless, involves a shift to a user-centric and community-centric perspective ^13^. CbICS are envisioned to comprise four levels of care for older adults that begin with ‘self-help’, followed by ‘informal support’ (including from family, neighbors, and community volunteers), then services provided through the long-term care insurance (LTCI) and health systems, and finally a safety net for those lacking other types of support, including poverty prevention measures. The distinctive emphasis is on the first two levels of care ^14, 15^, which in turn means that, rather than positioning public services as the primary providers of care, this role falls to older people themselves, together with their family members and the local community.

At the community level, increased citizen involvement enables the tailoring of services to local needs ^16^ as well as interventions that educate and help older adults to socialize so as to reduce frailty ^17^. Such activities, which blend formal and informal support, rest on an interface between local government organization and civic engagement ^18^. Generating the aimed-for voluntary participation and impact has, however, proven more challenging than expected ^10^. Another challenge that has been recognized in care is that well-meaning volunteers do not necessarily have the appropriate understanding of care practices to be effective ^19^. Thus, the broader community involvement envisioned as a prerequisite for the CbICS – in the form of volunteers who support neighbors and, more generally, enable the functioning of an inclusive local society – necessitates a sufficient understanding and knowledge of care, and the skills and capabilities to put these into practice. This need has been reinforced and highlighted throughout the COVID-19 pandemic. Thus, the development of the CbICS provides a unique, significant opportunity to understand the nexus of care, health and place for older adults, given the locally driven development of each CbICS.

## Developing the concept of ‘care literacy’

As the CbICS model includes formal health and care services as well as informal care in the family and community, care literacy is complementary to, yet distinct from, health literacy. Care literacy encompasses the knowledge and capabilities not only of the people in need of care, their carers and those involved in their health and care services, but also on the part of the citizenry as a whole; knowledge and capabilities that enable people in need of care to live their daily lives in the local community and enable potential care solutions. Thus, care literacy is important for ensuring that society as a whole offers a resilient and sustainable space for aging. Furthermore, the value of a broad understanding of care has been demonstrated by the need to adapt and re-adapt the delivery of health and care services during the ongoing COVID-19 pandemic. Across the community, the range of adaptations of care to the pandemic points to the importance and urgency of citizens’ understanding of care.

In such situations, given the complex interplay between provision of health and care issues, a consideration of health literacy is essential. Health literacy consists of three elements. The first is *functional literacy*, which concerns literacy in the most general sense, including the ability to read the relevant information about a medicine. Higher order health literacy ^20^ refers to aspects such as *communicative/interactive literacy*, which enables the communication of health information in every-day interactions with stakeholders, the sharing of information and the understanding of health situations, and *critical literacy*, which informs the critical understanding of information and its application to specific situations. Indeed, correlations have been noted between health information disparities and health outcomes ^21^, and such higher order health literacy has been emphasized to support empowerment with respect to health decision-making. In the context of care, corresponding higher order capabilities are required for the proactive involvement in care, which is complemented by a widespread understanding of the care needed for community engagement.

Thus, to complement the significant body of research on health literacy, there is a need to define and understand ‘care literacy’ and to trace the impact of ‘care literacy’ on older adults. Also, care literacy is not only needed to enable self-care, such as older adults supporting themselves, but also, critically, for local citizens to care for older adults, who could be a relative or a community member. Care literacy provides the understanding of how to adapt daily-life practices to meet the specific needs of an older person. Some practices will apply across diverse settings in everyday life, such as: hairdressers washing older customers’ hair at a salon for people with a degree of frailty, such as limited flexibility in the neck; appropriately helping older people to sit on and stand up from a park bench, not just by holding their hands; assisting a person in a wheelchair, such as helping them down a slope by walking backwards to avoid the sensation and risk of falling forward; and providing supermarket food shopping services to aid older people in achieving balanced nutrition, particularly for those living alone. Other practices are more distinctly location specific, for example: assisting with seasonal issues, such as snow shoveling in northern Japan; and subway etiquette in the metropolitan areas in order for all passengers to consider the presence of potentially vulnerable older people, such as attention to distances to keep, bag handling, and walking in a crowd, given the risks of, say, provoking a fall. Care literacy supports better care provision that contributes to health and well-being and enhances mitigation of risks from inappropriate or absent care practices, and enables an understanding of what underlies and motivates the variety of care practices. Thus, care literacy is about understanding the implications of care for others in the family and community, including the interplay of health and care issues, and the importance of place in understanding formal and informal local care delivery.

## ‘Care communication’: media and grassroot activities

To enhance care literacy, citizens need an understanding of care and related health issues and how these are addressed in their local area. Individual CbICS are being developed for each community, including ties, for instance, to local NPOs and associations, with the process of their respective development dependent on local priorities and resources. Thus, there is scope for grassroot processes that support citizen interaction with health and care professionals and staff to provide specialized information on care, integrated with a specific understanding of the evolving local CbICS. Further, the need to disseminate such information and knowledge within the broader community opens up a potential recognized critical role for media ^22, 23^, especially in response to events such as COVID-19, during which television has been rated the most credible amongst various media in Japan ^24^. Though limited by issues of reliability and credibility ^25^, social media also offers an avenue for citizen engagement and has been recognized to have the potential to contribute to health communications ^26^.

Such an informative role would also facilitate media’s distinctive capacity to scrutinize stakeholders and provide a voice to the most vulnerable ^22, 23^. Although CbICS enhance coordination and collaboration across services ^8^, bringing diverse providers closer together potentially attenuates the checks and balances enabled by more separate services, which raises the value of the independent scrutiny that media can provide. Also, care may place vulnerable people at risk, including care receivers and providers. The repercussions of the burdens and stress of care can be severe, at the extreme including violence, murder and suicide ^27, 28^. Furthermore, long-term involvement in care may alter life courses in ways not fully appreciated, which is a concern in some areas with young carers ^29^. Accordingly, the media may shed light on the vulnerabilities of the system and foster social discourse concerning key challenges in developing the CbICS and, more generally, adaptations to an aging society. To enable such an investigative and informative contribution, journalists would need an appropriate in-depth expertise and knowledge of care-related issues and for this to be, crucially, locally tailored. Access to relevant training, such as geriatrics and gerontology, would further enhance their credibility and thus impact. In other words, media specialization in care and aging should correspond to similar specializations in other areas of media interest, particularly in countries such as Japan, where super-aging will be a central social issue in the coming decades. Thus, important lines of enquiry are opened up by the potential significance of media in enabling development of care literacy that underpins the CbICS. 

## Conclusion

Care literacy refers to the knowledge and capabilities that enable people in need of care to live their daily lives in the community and society, and that facilitate potential health and care solutions: further understanding of care literacy and its impact is key to enabling older adults to achieve healthy aging in place. For Japan the need for care literacy stems from the advanced aging of society and the reshaping of care, to involve the whole community and coordinate the set of health and care services for older adults through development of individual CbICS in each local area. While aspects of care literacy are general and ubiquitous, other aspects are prominently local. Care literacy needs to include information on, and an understanding of, what care is needed and how care can be provided, which is specific to the local area. Furthermore, stepping back from a focus on Japan, there is significant evidence of cross-cultural differences in how people engage with care ^30^ as well as with healthcare in general ^31^. It follows that not only does care literacy need to be culturally nuanced, but the transition to citizen empowerment in the context of care will also differ across cultures and societies. At the same time, based on the CbICS experience in Japan, important commonalities are found in understanding the inter-connected domains of health and care, enabling anticipatory responses to evolving needs, and engaging all citizens in the creation of an inclusive community for older adults.

## Data availability

No data are associated with this article.
