# Supplementary material for: ‘Care literacy’ in super aging Japan
Source: Open Res Eur. 2021 Jul 27;1:85. [Version 1] doi: 10.12688/openreseurope.13853.1 (PMC10446049; doi:10.12688/openreseurope.13853.1)
Supplement: Peer review history

# Peer review: Reviewer response for version 1

**DOI:** 10.21956/openreseurope.14936.r27307

**Published:** 2021-09-06

**Authors:** Iza Kavedžija

**Affiliations:** 1 Department of Sociology, Philosophy and Anthropology, University of Exeter, Exeter, UK

This is a timely and well crafted essay presenting an idea of 'care literacy'. The particular case discussed, that of aging population in Japan and the emplacement of care in the community, is particularly relevant in the 'Decade of the Healthy Aging' announced by the UN. Japan offers a useful perspective not only due to advanced population aging, but also due to numerous developments in the field of community care.

 The essay opens up a range of further questions related to the implications of community based care. One of the questions emerging from exploration of the case of Japan regards the issue of responsibility placed on individual community members. What does a move towards care based in the community mean for the state, for families and for the individuals? What does the increasing tendency to promote community-based care mean for existing community organizations and NGOs working in the field of eldercare? In other words, are the limitations of community care largely due to 'illiteracy' of the community members, or are there systemic constraints that need to be addressed, too. 

 Overall, this is a valuable and stimulating article exploring a very important topic.

Is the argument persuasive and supported by appropriate evidence?

Yes

Is the work clearly and cogently presented?

Yes

Is the topic of the essay discussed accurately in the context of the current literature?

Yes

Does the essay contribute to the cultural, historical, social understanding of the field?

Yes

Reviewer Expertise:

Anthropology, Japan, Aging

I confirm that I have read this submission and believe that I have an appropriate level of expertise to confirm that it is of an acceptable scientific standard.

# Peer review: Reviewer response for version 1

**DOI:** 10.21956/openreseurope.14936.r27308

**Published:** 2021-08-17

**Authors:** Leng Leng Thang

**Affiliations:** 1 Department of Japanese Studies and Next Age Institute, National University of Singapore, Singapore, Singapore

It is delightful to learn from the paper the concept of ‘care literacy’ especially during such time of COVID-19 pandemic where vulnerable older individuals are faced with the risk of inadequate care needs in the community. Defined in the paper as the “knowledge and capabilities that enable people in need of care to live their daily lives in the community and facilitate potential health and care solutions,” the emphasis of *place* in the concept reinforces the important message of care integration as one that needs to include equipping the community to seriously enable one to aging-in-place with support. As what Coststantini, Nihei and Ueno state, ‘care’ encompasses more than the older adults who need care and their families, it also needs to be facilitated by those they come in contact with in everyday community life, including neighbours, shopkeepers, bus drivers and so on. They further provide in the paper a range of good examples about how care literacy will help the community and normal citizenry to adapt their practices keeping in mind appropriate care and support for the older persons in daily encounters and community life. Hence, the concept of ‘care literacy’ introduced has novelty for pushing beyond the normative notion of understanding care for the benefit of only the older individuals in need of care and their carers, but also including an emphasis essentially on community care literacy sensitized to local geographic specificities.

 Here, as this paper sets out to understand the value of care literacy, it has appropriately chosen to focus on how the concept would benefit community-based integrated care systems (CbICS) introduced since 2005 in super-aged Japan.

 The authors have provided a general understanding of CbICS which the Japanese government aims to establish in all local areas by 2025. The paper notes that CbICS adopts a user-centric and community centric perspective with four-levels of care, ranging from (1) the individual (self-help) and (2) family, neighbours, community volunteers (informal help), to formal support in (3) services from long-term care insurance and health system and (4) programs such as public assistance providing a safety net for those without other means of support. It is worthwhile noting the slight variation described in Sudo *et.al* (2018) on the first two levels, where the family is placed as a part of ‘self help’ with the individuals, and level 2 is referred to as ‘mutual aid’ provided through an informal network of volunteers. Either way, care literacy is focused on strengthening the first two levels and can be foreseen to play important role complementing health literacy to promote better well-being among community-dwelling older adults.

 In the last section, the paper explores practical ways to enhance care literacy through grassroots activities and the media. It will be ideal if everyone – regardless of whether there is a well-developed CbICS in place - to see the necessity to empower themselves through acquiring care literacy as a part of everyday community life. But in reality, it is common for people to only begin to pay attention to the health and care system around them when they are faced with individual needs or the needs of their loved ones. Given the challenge of the significant gap in public awareness and understanding among Japanese citizens on CbICS, the discussion in this section may also include further consideration of how the concept of ‘care literacy’ could interact with health literacy to attract one to want to learn about their local CbICS and their role to develop ‘care literacy’ along with health literacy. Besides media and grassroots activities, how can the whole of community endeavour, including school education efforts contribute to enhance care literacy in strengthening CbICS?

 I am excited with the potential of the emerging analytical concept of ‘care literacy’ in promoting an inclusive community. As the CbICS is developing at different rate in different localities, is there an example or two of local CbICS with community care practices close to actualizing the notion of ‘care literacy’? These could be convincing examples to show how adoption of the concept would bring new lens in rooting care in the community.

 In a way, the notion of ‘care literacy’ resonates well with the vision of an age-friendly and dementia-friendly community commonly discussed in the context of aging-in-place. It would be useful to see also some engagements with these existing frameworks in delineating the background situating ‘care literacy’ enabling aging in place.

Is the argument persuasive and supported by appropriate evidence?

Yes

Is the work clearly and cogently presented?

Yes

Is the topic of the essay discussed accurately in the context of the current literature?

Yes

Does the essay contribute to the cultural, historical, social understanding of the field?

Yes

Reviewer Expertise:

NA

I confirm that I have read this submission and believe that I have an appropriate level of expertise to confirm that it is of an acceptable scientific standard.

## References

[1] : Japan's healthcare policy for the elderly through the concepts of self-help (Ji-jo), mutual aid (Go-jo), social solidarity care (Kyo-jo), and governmental care (Ko-jo). *Biosci Trends* .2018;12(1) : 10.5582/bst.2017.01271 7-11 10.5582/bst.2017.01271 29479017
